# Is serum vitamin D levels associated with disability in patients with newly diagnosed multiple sclerosis?

**Published:** 2013

**Authors:** Hamidreza Hatamian, Elham Bidabadi, Seyed Mohammad Seyed Saadat, Niloufar Seyed Saadat, Ehsan Kazemnezhad, Hamed Ramezani, Babak Bakhshayesh

**Affiliations:** 1 Associate Professor, Department of Neurology, Poursina Hospital, Guilan University of Medical Sciences, Rasht, Iran; 2 Assistant Professor, Department of Pediatric Neurology, Guilan University of Medical Sciences, Rasht, Iran; 3 Medical Student, Medical Student Research Committee, Guilan University of Medical Sciences, Rasht, Iran; 4 Trauma Research Center, Guilan University of Medical Sciences, Rasht, Iran; 5 Resident, Department of Neurology, Poursina Hospital, Guilan University of Medical Sciences, Rasht, Iran; 6 Assistant Professor, Department of Neurology, Poursina Hospital, Guilan University of Medical Sciences, Rasht, Iran

**Keywords:** Multiple Sclerosis, Vitamin D, 25-hydroxyvitamin D, Deficiency

## Abstract

**Background:**

Although the precise etiology of multiple sclerosis (MS) is unknown, it seems that both genetic and environmental factors are important. Recent studies suggest that low serum vitamin D levels are important environmental factor in MS. The aim of this study was to compare the serum levels of vitamin D between MS patients and healthy subjects, and to determine its association with disability in MS patients.

**Methods:**

In this cross-sectional study, a total of 52 patients with MS were randomly recruited and matched for age and sex with 52 healthy subjects. Demographic characteristics and serum vitamin D levels for both groups, as well as duration of disease Expanded Disability Status Scale (EDSS) for MS patients were evaluated. Statistical analysis was performed by independent samples t-test and multiple linear regression analysis.

**Results:**

The mean serum vitamin D levels were 26.5 ± 16.3 ng/ml in MS patients vs. 37.1 ±19.7 in healthy subjects (P = 0.003). A linear regression analysis showed no significant association between vitamin D levels and EDSS score of patients with MS (P = 0.345), after adjusting for the covariates.

**Conclusion:**

Our findings did not suggest a protective association for serum vitamin D levels against disability in MS patients.

## Introduction

Multiple sclerosis (MS) is a chronic and disabling autoimmune disorder of the central nervous system with considerable social and economic impact.^1–3^ The most prevalent age of MS is between 20 and 40 years,^4^ and it affects women twice compared to men.^5^ MS usually has a relapsing-remitting (RR) course for about 10 years with phases of clinical disease followed by recovery.^2, 6^

The pathogenic mechanisms of MS are not fully understood.^6^ It seems that both genetic and environmental factors play a role.^7^ The reports of recent studies regarding the association of low serum vitamin D levels and MS have been somehow conflicting in different populations,^8–18^ and we need more confirmatory studies.^14, 19^ Data from recent studies indicate that 25-hydroxyvitamin D levels are lower in MS patients than healthy subjects,^8, 10, 12, 17^ but not in other studies.^9, 11, 13^ 25-hydroxyvitamin D has also been linked to relapsing rate, disability, and severity of illness in some studies.^6, 10, 20, 21^ In addition, high circulating levels of 25-hydroxyvitamin D have been associated with a lower risk of MS.^22^ High levels of 25-hydroxyvitamin D was associated with a high chance of remaining relapse-free and low Expanded Disability Status Scale (EDSS) scores in patients with relapsing-remitting MS (RRMS).^18, 21^

The prevalence and incidence of MS in Middle Eastern countries, especially in Iran, has recently been reported to be higher than what has previously been speculated.^23–29^ In Saudi Arabia, the reported prevalence has raised from 8 per 100,000 in 1977 to 25 per 100,000 in 1998.^24^ In Isfahan, Iran, the prevalence and incidence of MS have dramatically increased in recent years, as the estimated prevalence and incidence has increased from 43.8 and 3.64 person per 100,000 population, respectively in 200730 to 73.3 and 9.1 person per 100,000 population in 2009.^25^ This critical increase might be due to high prevalence of vitamin D deficiency among populations of the Middle Eastern countries and Iran.^25, 31–36^ Vitamin D deficiency seems to be a more serious problem in northern Iran than other parts of the country because of special geographic situation including higher latitude, greater humidity, greater distance from the Equator, and least sunlight exposure.^32, 37^ However, no reliable statistics on MS are available in northern Iran. Therefore, it is of great importance to conduct further studies regarding MS disease in this region.

Considering the moderate to high prevalence of MS in Iran,^25, 26, 30, 38^ and given that there has been few evidences in the literature about the relationship between serum vitamin D levels and MS disease in the Middle East,^17^ this study was designed to first compare the serum 25-dihydroxyvitamin D levels in MS patients with healthy subjects of northern Iran (Guilan province), and then determine its possible association with disability in patients with MS.

## Materials and Methods

### Study Design

This cross-sectional study was performed in the Neurology Department of Poursina Hospital, Guilan University of Medical Sciences, northern Iran. All the assessments were undertaken in the summer of 2010. A total of 52 patients of Guilan MS Society with RRMS confirmed by clinical findings and magnetic resonance imaging (McDonald criteria) were included in this study. Inclusion criteria were as follows: 1) EDSS score less than 5.5, 2) disease course of at least four months based on the first symptoms of the disease, and 3) being in the remission phase of MS without experiencing any new attack during the last 30 days.

To recruit volunteers of the control group, employed personnel's of Poursina Hospital interested to be freely evaluated for their serum vitamin D status, were called to participate in this study. Fifty two healthy subjects as controls were randomly selected among volunteer hospital staffs, and were matched for time and date of sampling, age and sex. People who were not born and did not reside in Rasht, had the history of taking vitamin D supplements within a year before the study, or had a disease affecting vitamin D levels were excluded.

### Data Collection

Healthy subjects and patients with MS who were registered in MS Society of Guilan were randomly invited to participate in this study. Written informed consent was obtained from all participants. Blood samples were obtained after overnight fasting. Demographic characteristics including age and gender, duration of disease from the first symptom presentation, relapse history, history of disease-modifying therapies (DMT), and EDSS were collected by two neurologists. When different EDSS scores were noted by each neurologist, their consensus was recorded. Blood samples were measured by chemiluminescent immunoassay method (DiaSorin Liaison kit, Italy) for 25-hydroxyvitamin D levels, all in the same laboratory.

### Statistical analysis

Statistical analyses were carried out using SPSS software, version 18.0 (SPSS, Inc., Chicago, IL, USA). Normality of variables was assessed by Kolmogorov-Smirnov test. Mean values and standard deviations of vitamin D levels in healthy subjects and patients with MS were compared using an independent samples t-test. The relationship between vitamin D levels and EDSS were determined using multiple linear regression analysis with an Enter approach for univariate analysis and backward-elimination, controlling for sex, age and disease duration for adjusted analysis. Adjusted R^2^ was used to determine the variance of EDSS. Moreover, Pearson's correlation analysis was used to determine the relationship between duration of disease and serum vitamin D levels. P-values < 0.05 were considered to be statistically significant.

### Geographic location of the study area

Rasht is the capital of Guilan province in northern Iran, and is the largest city along the Caspian Sea coast, located between 37° 00’ and 38° 27’ north latitude. It has a humid and unstable climate and its average annual humidity is approximately 81.2 percent. It has the highest rate of rainfall among other provinces in Iran with an annual rainfall of 1355.5 mms.

## Results

**Figure 1:**
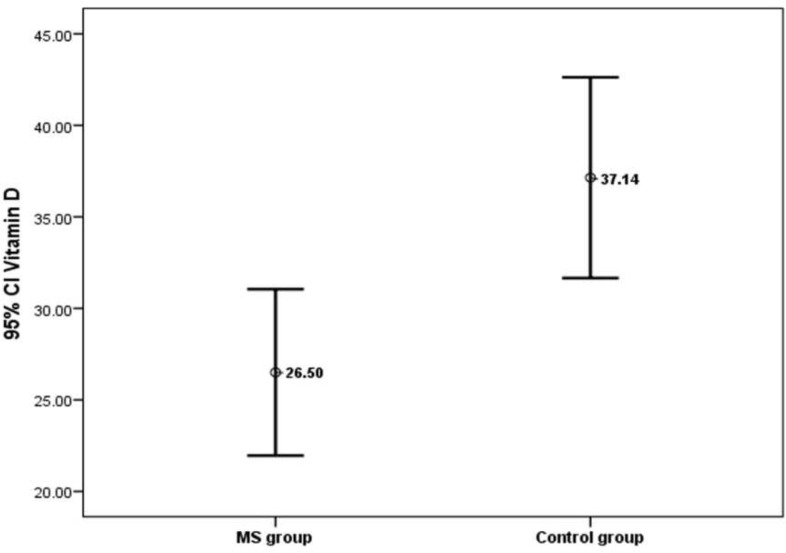
Mean vitamin D level in multiple sclerosis (MS) patients and healthy controls (P = 0.003)

A total of 52 MS patients were included in the study and matched with 52 healthy subjects as controls. The demographic and clinical characteristics of all participants are summarized in Table 1. Mean age was 28.4 ± 5.4 years for patients and 28.3 ± 4.5 years for controls (P = 0.953). Most of the participants (70% of MS patients and 74% of controls) were female. All MS patients were using Interferon-beta (INF-beta) as their DMT. No additional medications affecting serum vitamin D were used by patients. Mean EDSS score of MS patients was 3 ± 1.24 and mean duration of disease was 34.8 ± 20.0 months. The mean serum vitamin D levels were 26.5 ± 16.3 ng/ml in patients with MS vs. 37.1 ± 19.7 in the controls (P = 0.003) (Figure 1).

**Table 1:** Demographic and clinical characteristics of the study subjects

	Multiple sclerosis patients (n = 52)	Healthy controls (n = 52)	P
Female (%)	38 (73.1%)	36 (69.2%)	0.829
Age (year)	28.4 ± 5.4	28.3 ± 4.5	0.953
Vitamin D (ng/dl)	26.5 ± 16.3	37.1 ± 19.7	0.003
Disease duration (month)	34.8 ± 20.0	-	-
EDSS	3.0 ± 1.2	-	-

EDSS: Expanded Disability Status Scale

Numerical values are presented as Mean ± SD

Univariate analysis revealed that vitamin D (β = -0.01, P = 0.34), sex (β = 0.27, P = 0.45) and age (β = 0.025, P = 0.42) were not significantly associated with EDSS. However, duration of disease (β = 0.02, P = 0.02) was a significant predictor of EDSS (Table 2). Additionally, an inverse correlation was observed between serum vitamin D levels and duration of disease (r = -0.280, P =0.044).

**Table 2:** Univariate relationships between independent variables and Expanded Disability Status Scale

	β coefficient	P	Standard error
Sex	0.27	0.45	0.36
Age	0.25	0.42	0.03
Duration of disease	0.02	0.02	0.009
Vitamin D	-0.01	0.34	0.01

In a backward linear regression analysis including all variables of univariate analysis, no significant association was found between vitamin D levels and EDSS controlling for sex, age and duration of disease. However, duration of disease was the only significant predictor for EDSS (β = 0.023, P = 0.007, Adjusted R^2^ = 0.119), as the EDSS linearly increased by 0.023 per each year increase in duration of disease.

A full adjusted regression model explained 10.5% of the variance of EDSS. We used the backward-elimination method to evaluate the contribution of vitamin D and adjusted covariates in explaining variance of EDSS (Table 1). We included vitamin D, duration of disease, sex, and age in the model; which explained 10.5% of the total variance of EDSS in the first step. In the second step, which sexwas excluded, 11.3% of the variance for dependent variables was explained. In the third step, the remaining two variables including vitamin D and duration of disease accounted for an additional 12.2% of the variance and in the final step, duration of disease explained 11.9% of the variability in EDSS, and was the only significant contributor to EDSS. The overall model was significant (EDSS = 0.023 duration of disease + 2.2; r = 370, P = 0.007)

## Discussion

Findings of the current study suggested that vitamin D levels in patients with MS were significantly lower than the control group, especially in women. In addition, serum vitamin D level was not associated with disability, after adjusting for other variables. However, duration of disease, as had previously been reported,^39^ was significantly related with disability.

Findings of study had some similarities and differences to what have been reported in the literatures to date. Correale et al.^12^ assessed serum 25-hydroxyvitamin D levels between Spanish MS patients and healthy subjects of control group. They observed a significantly lower 25-hydroxyvitamin D levels in relapsing remitting patients (47.3 ± 9.0 pg/ml during remission, and 38.5± 8.7 pg/ml during exacerbations) compared to healthy subjects (61.2 ± 5.6 pg/ml). In contrast, Kragt et al.^13^ performed a large prospective cohort with 101 patients and 107 controls in the Netherlands; patients with MS were followed during one year with vitamin D measurements (25 (OH) D and 1,25-dihydroxyvitamin D) in summer and winter. No differences in vitamin D levels were found between patients with MS and healthy individuals. Remarkably, the significant correlation between winter serum 25-hydroxyvitamin D levels and EDSS in a heterogeneous sample of Dutch patients with MS was restricted to women.

Additional studies reported a negative correlation between serum vitamin D levels and disability.^10, 18, 21^ In the study by van der Mei et al.^10^ that was performed on 136 MS patents and 272 community controls in the Tasmania, Australia, cases with higher disability (EDSS > 3) suffered from greater vitamin D insufficiency than cases with low disability. Smolders et al.,^21^ also reported a significant association between lower serum concentration of 25-dihydroxyvitamin D and higher EDSS in 267 patients. In 2012, Amini Harandi and colleagues,^18^ who investigated the association between serum vitamin concentration and EDSS score among 78 Iranian individuals with RRMS, showed a similar significant inverse correlation only in women. However, Yildiz et al.^40^ demonstrated no significant difference in serum vitamin D and EDSS between Swiss MS patients with high and low disease activity.

Contrary to some studies^9, 11, 13^ which the levels of vitamin D were not significantly lower in case group than healthy subjects, our findings revealed a significant difference in 25-dihydroxyvitamin D levels between MS patients compared to control group. Genetic variation in vitamin-D-related genes worldwide and different inclusion criteria are potential factors that could account for different findings between these studies. In addition, these differences may be due to unmatched groups in Kragt et al.^13^ study, low recruited participants in Soilu-Hanninen et al.^11^ and Barnes et al.^9^ study, and also non-fasting sampling and different methods for measuring serum vitamin D in Barnes et al.^9^ study.

In contrast to most previous studies^10, 21, 41^ which showed an inverse significant association between serum vitamin D and disability; we did not find such association. Low sample size in our study and differences in adjusted covariates, skin type variation, geographic situation, socioeconomic status, and different genetic background in various study populations might explain these different results. Another possible explanation is that we only included patients with a more recent onset of disease who suffered from a lower disability and received DMT for a fewer duration than similar previous studies. Thus, it is possible that the negative correlation between vitamin D levels and disability in MS patients found in previous studies was due to decreased outdoor activity of patients or their mood changes with the progression of disease. However, according to some limitations of the current study (smaller sample size), this finding should be interpreted with caution. Further studies focused on the relationship between serum vitamin D levels and disability in newly diagnosed MS patients is suggested. Moreover, measurements in different centers are influenced by regional variations in the general population, whereas differences in assay type probably have minor effects.^6^

Significant association of longer disease duration with worse EDSS score and also inverse relationship between vitamin D and duration of disease in our study may explain the cause of lower levels of vitamin D in MS group compared with healthy individuals. Because patients with prolonged disease duration have a higher disability,^39^ they might be expected to have less outdoor activities than normal population; therefore, they are at risk for vitamin D deficiency in long term because of poor sunlight exposure.^14^ It seems that because of cloudy weather in autumn and winter and also due to the high humidity in spring and summer in Guilan, people tend to have fewer outdoor activities; as a result, they are less exposed to sunlight.

According to results of our study, our model can predict a small percent of disability burden among MS patients; therefore, there are probably many other factors that might influence MS occurrence and disability.

Some limitations of the current study should be considered. Blood sampling in summer was the first limitation. The second limitation is the cross-sectional design, so causal relationship cannot be established; indeed vitamin D deficiency in patients with MS may be due to less exposure of MS patients to sunlight because of their illness rather than a causal relationship. In addition, we did not include patients with EDSS greater than 5.5 to our study assuming that they were subjects with poor sunlight exposure due to their disability and immobility. Slightly significant lower vitamin D levels in patients with longer illness duration in our study could indicate lower cumulative sun exposure in this population. On the other hand, our control group was selected from hospital staffs. This may have caused selection bias by itself due to a probable different knowledge of healthy participants about vitamin D deficiency and also their different life style and diet pattern. Therefore, we are not able to generalize this result to the general population and definitely conclude that subjects with MS have a lower vitamin D level. Further studies on the current topic are recommended especially in the Middle East. At last, although we did not find a significant correlation between low levels of vitamin D and EDSS, the hypothesis of the presence of such a correlation cannot be strongly denied. A larger prospective study evaluating MS patients for both serial serum vitamin D and EDSS is a more direct and statistically powerful way to determine whether low serum vitamin D is independently associated with EDSS.

Therefore, large cohort studies should be conducted in the future in order to follow people with various vitamin D levels and to evaluate the relative risk of MS development. MS is also associated with the higher risk of fracture and lower bone mass. Moreover, vitamin D insufficiency plays a substantial role in the risk of falls and fractures.^10, 42^ Furthermore, assuming a possible beneficial role of vitamin D in MS patients, and according to recent pilot safety and tolerability trials,^43–45^ we have come to a conclusion that large clinical trials should be conducted in the future to evaluate the safety and efficacy of different vitamin D analogues and doses in prophylaxis and treatment of MS.

In conclusion, vitamin D level in patients with MS was significantly lower than the healthy subjects but no significant relationship was found between vitamin D level and disability.
